# Weight gain during twin pregnancy with favorable pregnancy outcomes in Japan: A retrospective investigation for new criteria based on perinatal registry data

**DOI:** 10.1371/journal.pone.0253596

**Published:** 2021-07-02

**Authors:** Soichiro Obata, Mai Shimura, Toshihiro Misumi, Sayuri Nakanishi, Ryosuke Shindo, Etsuko Miyagi, Shigeru Aoki

**Affiliations:** 1 Perinatal Center for Maternity and Neonates, Yokohama City University Medical Center, Yokohama, Japan; 2 Department of Biostatistics, Yokohama City University School of Medicine, Yokohama, Japan; 3 Department of Obstetrics and Gynecology, Yokohama City University Hospital, Yokohama, Japan; University of Mississippi Medical Center, UNITED STATES

## Abstract

In 2009, the United States Institute of Medicine (IOM) reported the optimal gestational weight gain (GWG) during twin pregnancy based on the pre-pregnancy body mass index (BMI). However, there are ethnic variations in the relationship between GWG and pregnancy outcomes. We aimed to establish the criteria for optimal GWG during twin pregnancy in Japan. The study included cases of dichorionic diamniotic twin pregnancy registered in the Japan Society of Obstetrics and Gynecology Successive Pregnancy Birth Registry System between 2013 and 2017. We analyzed data for cases wherein both babies were appropriate for gestational age and delivered at term. Cases were classified into four groups based on the pre-pregnancy BMI: underweight (BMI <18.5 kg/m^2^), normal weight (18.5 kg/m^2^ ≤BMI< 25.0 kg/m^2^), overweight (25.0 kg/m^2^ ≤BMI< 30.0 kg/m^2^), and obese (BMI ≥30.0 kg/m^2^) and we calculated the 25^th^–75^th^ percentile range for GWG for the cases. The 3,936 cases were included. The GWG ranges were 11.5–16.5 kg, 10.3–16.0 kg, 6.9–14.7 kg, and 2.2–11.7 kg in the underweight, normal weight, overweight, and obese groups, respectively. Thus, in the current study, the optimal GWG during twin pregnancy was lower than that specified by the IOM criteria. Factoring this in maternal management may improve the outcomes of twin pregnancies in Japan.

## Introduction

Insufficient gestational weight gain (GWG) during pregnancy leads to an increase in the rates of preterm delivery and small for gestational age (SGA) births [1,2]. In contrast, excessive GWG is associated with increased rates of cesarean delivery, macrosomia [1,2], gestational diabetes mellitus (GDM) [3,4], and gestational hypertension (GH) [5–7]. In 2009, the United States Institute of Medicine (IOM) reported that the optimal GWG for twin pregnancy based on the pre-pregnancy body mass index (BMI) was as follows: 16.8–24.5 kg for normal weight (18.5 kg/m^2^ ≤BMI< 25.0 kg/m^2^), 14.1–22.7 kg for overweight (25.0 kg/m^2^ ≤BMI< 30.0 kg/m^2^), and 11.3–19.1 kg for obese (BMI ≥30.0 kg/m^2^) categories [8]. However, this optimal GWG was calculated based on the 25^th^–75^th^ percentile of the GWG in women who delivered twin babies at term (>36 weeks of gestation) in the United States and whose babies’ average birthweight was over 2,500 g [8]. Furthermore, previous studies have reported that there are ethnic variations in the relationship between GWG and pregnancy outcomes [9–11]. Therefore, this optimal GWG may not be directly applicable to women in Japan.

Given that GWG for most women with twin pregnancy in Japan appears insufficient on the basis of the IOM criteria [12], the optimal GWG for twin pregnancy in Japan may actually be less than that specified by IOM. However, no evidence-based studies have investigated the optimal GWG during twin pregnancy in Japan. Therefore, to establish the optimal GWG during twin pregnancy in Japan, we examined GWG during twin pregnancy in women with favorable pregnancy outcomes using the data from Japan Society of Obstetrics and Gynecology (JSOG) Successive Pregnancy Birth Registry System, the largest perinatal database in Japan.

## Materials and methods

Launched in 2001, the perinatal JSOG database includes delivery data obtained on or after 22 weeks of gestation from participating facilities throughout Japan. The number of participating facilities increased from 299 in 2013 to 396 in 2017, and 236,475 deliveries were registered in this database in 2017, representing 25.0% of all deliveries in Japan for that year. In this retrospective study, we selected the cases of dichorionic diamniotic (DD) twins registered in the perinatal JSOG database between 2013 and 2017. Cases involving still births, chronic hypertension, diabetes mellitus, GH, GDM, insufficient data, and outliers (maternal height outside the range of 140 to 180 cm, maternal weight outside the range of 30 to 120 kg or birthweight <400 g) were excluded. From the remaining cases, we selected those in which both babies were appropriate for gestational age (AGA) and delivered on or after 34 weeks of gestation.

The included cases were classified into the following four groups based on their pre-pregnancy BMI: 1) underweight (BMI<18.5 kg/m^2^), 2) normal weight (18.5 kg/m^2^ ≤BMI< 25.0 kg/m^2^), 3) overweight (25.0 kg/m^2^ ≤ BMI< 30.0 kg/m^2^), and obese (BMI ≥30.0 kg/m^2^). In addition, from these cases, cases that were delivered at term (after 36 weeks of gestation) were extracted. We examined the maternal background characteristics (age, height, pre-pregnancy bodyweight, bodyweight at delivery, and rates of primipara) and pregnancy outcomes (gestational weeks at delivery and average birthweight of both babies).

During the study period, GDM was defined as follows: fasting plasma glucose ≥92 mg/dL and 1-h value ≥180 mg/dL or 2-h value ≥153 mg/dL on the 75-g oral glucose tolerance test. GH was defined as hypertension (systolic blood pressure ≥140 mmHg and/or diastolic blood pressure ≥90 mmHg) at and following 20 weeks of gestation. The database does not include data on the socioeconomic status, nutritional status, and environmental exposures; thus, we could not analyze these factors.

The present study was approved by the ethics committee of the Yokohama City University Medical Center (B190600057), and all methods were performed in accordance with the relevant guidelines and regulations. Because this study used an anonymized database, we could not obtain informed consent from the participating individuals; although the ethics committee decided that informed consent was not required due to the retrospective nature of the study, we utilized the opt-out method.

### Statistical analysis

JMP Pro (version 15.0, SAS Institute, Cary, NC, USA) was used for all statistical analyses. Maternal background data and pregnancy outcomes were presented as medians and ranges or as numbers and percentages. The Kruskal–Wallis test was used for analyzing continuous variables, while the Pearson’s chi-squared test was used for analyzing categorical variables. The level of statistical significance was set at P < 0.05. In addition, we calculated the 25^th^–75^th^ percentile range for GWG in each group.

Therefore, our primary endpoint was examining the GWG ranges in DD twin cases with favorable pregnancy outcomes in Japan by calculating the 25^th^–75^th^ percentiles of the GWG in women with DD twins (both AGA at term (after 36 weeks of gestation]) that were included in the perinatal JSOG database. Our secondary endpoint was the difference between the GWG during twin pregnancy in women with favorable pregnancy outcomes and the optimal GWG specified by the IOM.

## Results

**Fig 1:**
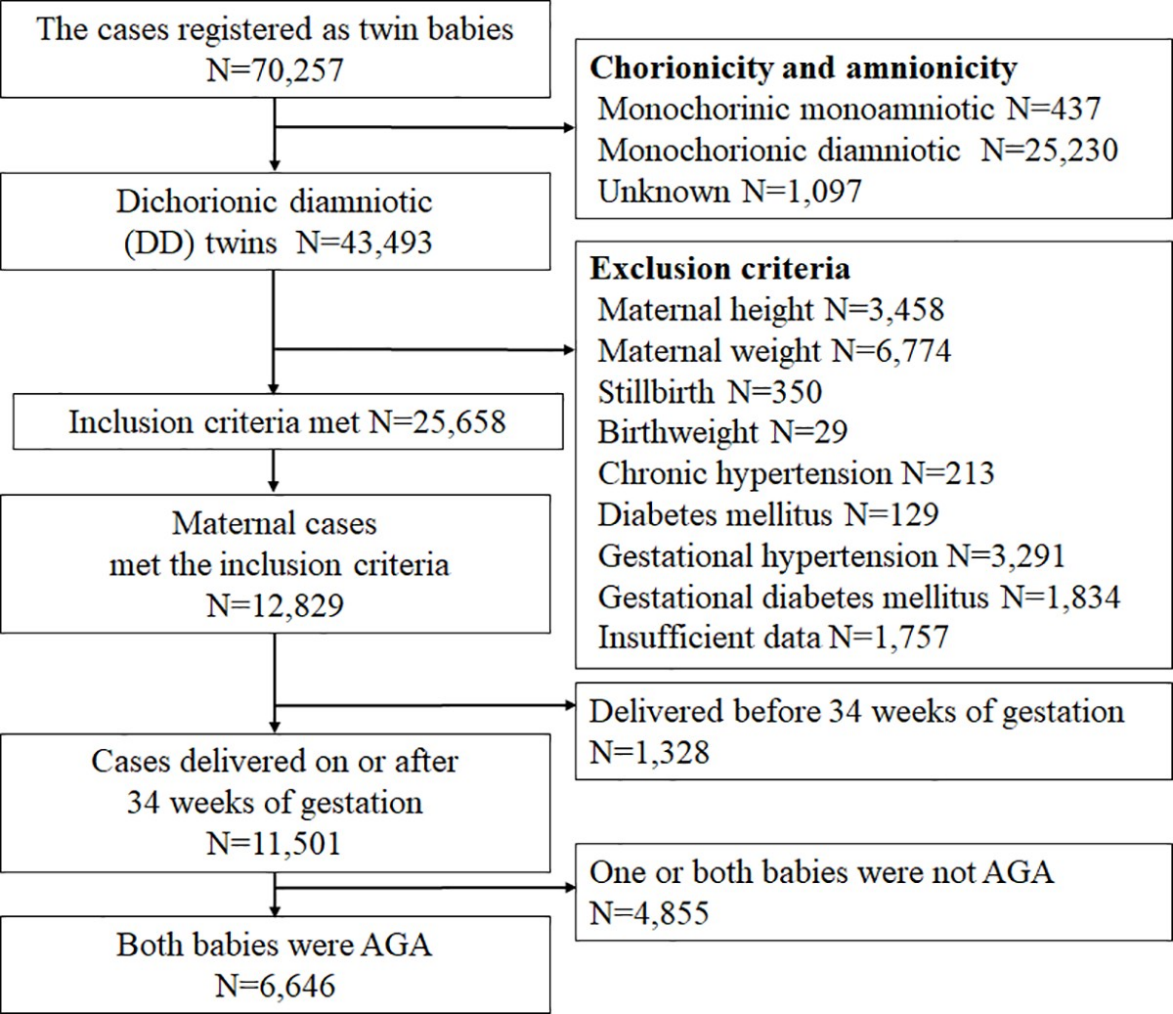
Flowchart depicting the extraction of the eligible cases from the JSOG database. Each newborn is registered in the perinatal JSOG database. A total of 70,257 newborns were registered as twins. Of these, 25,658 newborn cases met the inclusion criteria and were included. We selected the cases wherein both babies were AGA and delivered on or after 34 weeks of gestation; accordingly, a total of 6,646 women with DD twin pregnancy were included.

**Fig 2:**
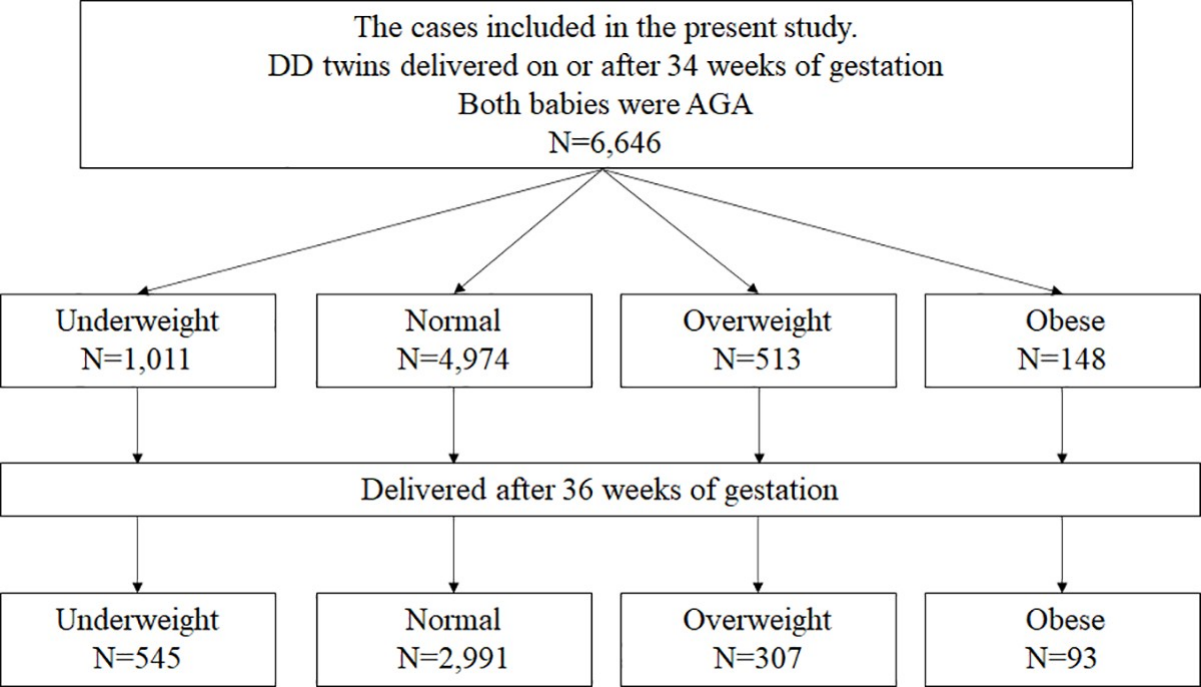
Flow chart of the cases included in the present study. Underweight (BMI <18.5 kg/m^2^), normal weight (18.5 kg/m^2^ ≤ BMI < 25.0 kg/m^2^), overweight (25.0 kg/m^2^ ≤ BMI < 30.0 kg/m^2^, obese (BMI ≥ 30.0 kg/m^2^). The number of cases refers to the number of women who delivered the twins. BMI: Body mass index.

The flow chart of cases is presented in Figs 1 and 2. Each newborn is registered in the perinatal JSOG database. A total of 70,257 newborns were registered as twins. Of these, 25,658 newborn cases and 12,829 women with twin pregnancy met the inclusion criteria. We selected the cases where both babies were AGA and delivered on or after 34 weeks of gestation. Accordingly, data for 6,646 women with twin pregnancy were analyzed. According to the BMIs, 1,011, 4,974, 513, and 148 women were classified into the underweight, normal weight, overweight, and obese groups, respectively. Of these, infants were delivered at term for 545, 2,991, 307, and 93 women, respectively.

The background characteristics and pregnancy outcomes of the cases are shown in Table 1. The average birthweights tended to increase as the pre-pregnancy BMI increased. An optimal GWG, based on the IOM criteria, was noted in only 18.5–25.7% of the cases with favorable outcomes (i.e., delivery of both AGA babies after 36 weeks of gestation). The GWG was lower than the optimal IOM value in over 70% of the cases in each group. Excessive GWG, based on the IOM criteria, was observed in 5.4% of the cases in the obese group and in under 2% of the cases in the normal weight and overweight groups.

**Table 1:** Maternal background characteristics and pregnancy outcomes for cases in which both twins were classified as appropriate for gestational age.

	Underweight (N = 545)	Normal weight (N = 2,991)	Overweight (N = 307)	Obese (N = 93)	P Value
Age (Year)	33 (20–51)	33 (14–53)	32 (17–46)	33 (25–45)	0.067
Primipara	344 (63.1)	1,624 (54.3)	167 (54.4)	44 (47.3)	< .001
Height (cm)	161 (145–175)	160 (143–178)	159 (147–180)	158 (148–172)	< .001
Pre-pregnancy body weight (kg)	46.0 (35.3–55.4)	53.0 (41.0–75.0)	67.0 (55.0–91.9)	81.0 (68.0–110.0)	< .001
Body weight at delivery (kg)	60.0 (37.4–81.2)	66.2 (39.9–101.4)	77.8 (43.0–105.8)	89.6 (58.6–111.6)	< .001
Adequate GWG based on the IOM criteria	N/A	553 (18.5)	79 (25.7)	20 (21.5)	< .001
Insufficient GWG based on the IOM criteria	N/A	2414 (80.7)	222 (72.3)	68 (73.1)	
Excessive GWG based on the IOM criteria	N/A	24 (0.8)	6 (2.0)	5 (5.4)	
Gestational age at delivery (weeks)	37.3 (37.0–39.6)	37.3 (37.0–40.4)	37.4 (37.0–40.3)	37.3 (37.0–38.7)	0.622
Average birthweight for both twins (g)	2,553 (2,180–3,230)	2,575 (2,179–3,293)	2,638 (2,219–3,179)	2,645 (2,290–3,018)	< .001

Data are presented as numbers (percentage) or medians (range).

Underweight (BMI <18.5 kg/m^2^), normal weight (18.5 kg/m^2^ ≤ BMI < 25.0 kg/m^2^), overweight (25.0 kg/m^2^ ≤ BMI < 30.0 kg/m^2^, and obese (BMI ≥ 30.0 kg/m^2^).

AGA: Appropriate for gestational age; IOM: Institute of Medicine; GWG: Gestational weight gain; BMI: Body mass index.

The IOM criteria do not specify the optimal GWG for the underweight group.

Table 2 shows the 25^th^–75^th^ percentile for GWG for cases in each BMI category in which both babies were AGA. The GWG among the cases with favorable outcomes (i.e., delivery of two AGA babies after 36 weeks of gestation) ranged 11.5–16.5 kg, 10.3–16.0 kg, 6.9–14.7 kg, and 2.2–11.7 kg and the GWG among the cases delivered on or after 34 weeks of gestation ranged from 10.4–15.7 kg, 9.6–15.2 kg, 6.7–14.0 kg, and 1.2–10.4 kg in the underweight, normal weight (IOM criteria: 16.8–22.7 kg), overweight (IOM criteria: 14.1–22.7 kg), and obese (IOM criteria: 11.3–19.1 kg) groups, respectively. The optimal GWG tended to decrease as the BMI increased, and the ranges were much lower than those specified in the IOM criteria.

**Table 2:** Gestational weight gain among the cases with both babies appropriate for gestational age.

	Underweight	Normal weight	Overweight	Obese	P-Value
GWG among the cases with favorable outcomes (kg)	14.0 (11.5–16.5)	13.0 (10.3–16.0)	10.5 (6.9–14.7)	6.2 (2.2–11.7)	< .001
GWG among the cases delivered on or after 34 weeks of gestation (kg)	13.0 (10.4–15.7)	12.4 (9.6–15.2)	10.1 (6.7–14.0)	6.0 (1.2–10.4)	< .001

Data are presented as median (25^th^-75^th^ percentile).

Underweight (BMI < 18.5 kg/m^2^), normal weight (18.5 kg/m^2^ ≤ BMI < 25.0 kg/m^2^), overweight (25.0 kg/m^2^ ≤ BMI < 30.0 kg/m^2^, and obese (BMI ≥ 30.0 kg/m^2^).

BMI: Body mass index.

A favorable outcome was defined as delivery after 36 weeks of gestation and both twins appropriate for gestational age.

## Discussion

In the present study, we aimed to examine the GWG criteria for women with twin pregnancy with favorable pregnancy outcomes in Japan. Our findings indicated that the GWG among women with favorable outcomes (i.e., delivery of two AGA babies after 36 weeks of gestation) was below the optimal IOM-specified values in over 70% of the cases with favorable outcomes registered in the perinatal JSOG database and that IOM criteria were met only 18–25% of the cases. In addition, in each BMI group, the ranges for the 25^th^–75^th^ percentile of GWG among cases with favorable outcomes were much lower than those specified in the IOM criteria.

Previous studies have reported ethnic variations in the GWG and pregnancy outcomes such as birthweight [9–11]. One previous study noted that the GWG among women in Japan tended to be lower than that among women in the United States [13]. Moreover, a single-center report by Suzuki noted that GWG tended to fall below the optimal IOM value in a large percentage of twin pregnancies in Japan [12]. Thus, our findings are in accordance with previous data related to optimal GWG for twin pregnancy.

In the present study, we calculated the 25^th^–75^th^ percentile ranges for GWG among cases with favorable outcomes. In addition, because the rate of preterm delivery in twin pregnancy is high, we examined the cases delivered on or after 34 weeks of gestation. Ranges in the latter three BMI groups were lower than those specified in the IOM criteria; the IOM criteria do not specify the optimal GWG for the underweight group due to insufficient evidence [8]. This finding highlights the need to consider differences in the physical size and ethnicity when applying the IOM criteria, given that women in Japan are generally smaller than those in the United States, and therefore, the optimal level of GWG may be lower for women in Japan than in the United States. In their analysis of perinatal data from the JSOG database, Morisaki et al. reported that the optimal GWG for singleton pregnancy in Japan was 12.2 kg, 7.7–10.9 kg, and 4.3 kg for women with BMIs of 17–18.4 kg/m^2^, 18.5–24.9 kg/m^2^, and 25.0–27.4 kg/m^2^, respectively [14]. The higher values observed in the present study are reasonable given that optimal GWG during twin pregnancy should be higher than that during singleton pregnancy. Consistent with our findings, the single-center study by Suzuki reported that the GWG were 16.0±2.4 kg, 13.9±3.4 kg, 10.2±4.8 kg, and 7.9±3.4 kg in the underweight, normal weight, overweight, and obese groups, respectively [12]. Furthermore, several previous studies have indicated that the optimal GWG decreases as the pre-pregnancy BMI increases [2,8,12,15–17], which is also consistent with our results.

As mentioned above, the IOM criteria do not specify the optimal GWG for the underweight group due to insufficient evidence [8]. In Japan, the number of women included in the underweight group is higher than that in the United States. Enomoto et al. investigated the GWG during singleton pregnancy using the same perinatal JSOG database, reporting that 18.2%, 7.7%, and 2.9% of the women fell within the underweight, overweight, and obese groups [18], respectively. The high percentage of women in the underweight group highlights the need to investigate the optimal GWG among these individuals. Of the 6,646 cases included in the present study, 1,011 (15.2%) were included in the underweight group. To our knowledge, the present study represents the largest analysis of GWG during twin pregnancy among underweight women in Japan. Our findings indicated that the GWG among the twin pregnancy cases with favorable outcomes in the underweight group ranged from 11.5 to 16.5 kg, which is higher than that observed among other groups in our study and among women with singleton pregnancies in Japan. In addition, the range observed in the present study is lower than those specified in the IOM criteria for other groups. Therefore, we consider this GWG range to be reasonable and reliable.

The present study possesses several limitations of note. First, because we used the perinatal JSOG database, we were unable to include cases in which delivery occurred at non-participating facilities, and we were unable to exclude the influence of input errors. Second, the overweight and obese groups included relatively low numbers of women. Third, because we calculated optimal GWG based on gestational age at delivery and birthweight, we could not fully consider the influence of GWG on GH or GDM. Moreover, we examined optimal GWG for cases with favorable outcomes only. Despite these limitations, the results of the present study are important given that we analyzed data from the largest perinatal registry system in Japan.

## Conclusions

We show that the GWG range women with DD twin pregnancies with favorable outcomes in Japan is reasonably lower than the IOM-specified ranges. Because we excluded cases of complicated with GH and GDM, future studies should investigate the influence of GDM or GH on the optimal GWG during twin pregnancy. Prospective studies are required to verify the validity of our findings regarding the GWGs in this population.
